# *Neospora caninum*: Recent Progress in Host-Pathogen Interactions, Molecular Insights, and Control Strategies

**DOI:** 10.3390/microorganisms14020338

**Published:** 2026-02-02

**Authors:** Karim Debache, Andrew Hemphill

**Affiliations:** 1 Debache Switzerland, Heimstrasse 70, 3018 Bern, Switzerland; 2 Institute of Parasitology, Vetsuisse Faculty, University of Bern, Länggass-Strasse 122, 3012 Bern, Switzerland

**Keywords:** apicomplexa, *Neospora caninum*, vertical transmission, virulence, diagnosis, epidemiology, CRISPR/Cas9, vaccine, drug targets

## Abstract

*Neospora caninum*, the causative agent of abortion in cattle, has a major economic impact worldwide. This review aims to provide an overview of key advances over the last 10 years in understanding host−pathogen interactions, molecular mechanisms, and emerging control strategies and puts them into a context with previously published important findings. More recently, novel diagnostic tools with improved sensitivity and specificity have been developed. These have supplemented the already existing methods to detect infection in clinical cases and are essential for investigations on parasite distribution, disease incidence and prevalence, and transmission of *N. caninum.* Epidemiological studies have revealed the influence of environmental, genetic, and ecological factors on parasite transmission dynamics, and emphasized the importance of integrated “One Health” strategies. Characteristics of different *Neospora* strains have been elucidated through animal models and molecular tools such as clustered regularly interspaced short palindromic repeats/CRISPR associated protein 9 (CRISPR/Cas9)-based gene editing, high-throughput sequencing, and advanced proteomics, aiming to shed light on stage-specific gene regulation and virulence factors, contributing to the development of interventions against neosporosis. Insights into immune modulation, immune evasion, and parasite persistence contributed to the efforts towards vaccine development. In terms of therapeutics, both repurposed drugs and more targeted inhibitors have shown promising efficacy in reducing parasite burden and mitigating vertical transmission in laboratory models. Here, more recent innovations in nanoparticle-based drug delivery systems and immunomodulatory strategies are prone to enhancing therapeutic outcomes. However, a significant challenge remains the integration of molecular and immunological insights into practical applications.

## 1. *Neospora caninum* and Neosporosis

*Neospora caninum* is an apicomplexan parasite that was isolated from dogs with encephalomyelitis and myositis [1]. After its discovery, it has emerged as a significant pathogen causing abortion and stillbirth in cattle and neurological disorders in dogs. Canids such as dogs, coyotes, gray wolves, and dingoes act as definitive hosts [2,3], while with respect to intermediate hosts *N. caninum* infects a diverse range of other farm animals and wildlife species [4,5,6]. This broad host range contributes to parasite persistence in various ecosystems and complicates control efforts.

The life cycle of *N. caninum* includes three distinct invasive stages. The rapidly proliferating tachyzoite stage causes acute disease and can invade and replicate in many tissues and cell types [7]. In vitro culture allows the visualization of tachyzoites, their interactions with the host cells, and their intracellular development by different microscopical techniques. Surface antigens (SAGs) mediate the first contact between host cell surface and parasite membrane, closely followed by the secretion of three types of secretory organelles crucially involved in host cell invasion. Initially, micronemes and rhoptries sequentially secrete MIC and ROP proteins, respectively, through the apical tip of the parasites. MIC and ROP proteins interact with host cell surface receptors and are involved in the formation of a gliding junction, which allows the parasite to invade by pushing the host cell surface membrane into the cytoplasm, until the parasite is located intracellularly and proliferates within a parasitophorous vacuole (PV), delineated by a parasitophorous vacuole membrane (PVM) [2,3].

Host cell invasion is followed by the secretion of dense granule (GRA) proteins into the lumen of the PV, some of which form the intravacuolar tubular network, modify the PVM or cross the PVM and enter the host cell nucleus to act as transcription factors. Several MIC, ROP, and GRA proteins constitute virulence factors and have been exploited as vaccine candidates [7,8]. In an immunocompetent host, tachyzoites will differentiate into slowly replicating bradyzoites that form tissue cysts and cause chronic infection. Tissue cysts are found mainly in the central nervous system (CNS) and in muscle tissue without causing symptoms. They are orally infectious for a carnivorous host. The third invasive stage is the sporozoites that are formed in the environment following the sexual development and oocyst formation that takes place in the intestinal tissues of the definitive host.

The life cycle of *N. caninum* starts by accidental uptake of infective oocysts containing sporozoites through contaminated food or water or ingestion of meat containing *N. caninum* tissue cysts formed by bradyzoites. These stages lead to the infection of enterocytes and proliferation within intestinal epithelial cells. Parasites multiply and undergo egress, followed by invasion of cells of the reticulo-endothelial system, including antigen-presenting dendritic cells, in which they further disseminate into other locations and tissues in the body as tachyzoites [9]. Infection goes largely unnoticed, but the inflammatory immune response eliminates a large part of the parasite population. The associated physiological stress triggers tachyzoite-to-bradyzoite differentiation in a subset of these parasites, resulting in the formation of tissue cysts, which finally persists mainly in the CNS and in skeletal muscle [10]. Ingestion of tissues containing bradyzoites by a definitive host can then lead to sexual development and oocyst formation in the intestine [6].

In pregnant animals, tachyzoites can be vertically transmitted and infect the fetus. Vertical transmission is highly relevant in cattle but has been reported also in several other animal species [6,8]. Transplacental vertical transmission can occur through the exogenous route following a primary infection during pregnancy or endogenously in chronically infected animals upon recrudescence of bradyzoites and conversion into tachyzoites during pregnancy. In both cases, parasites exploit the pregnancy-associated impairment of inflammatory immunity normally serving to protect the developing fetus, while at the same time increasing the risk of fetal infection. Vertical transmission rates vary significantly between host species [6,7], with fetal infection often resulting in abortion [11]. In intermediate hosts, particularly cattle, endogenous transplacental transmission can occur repeatedly through successive pregnancies. This process of interconversion between developmental stages is crucial for both parasite persistence and transmission, involving complex immunological interactions during both acute and chronic phases of infection [12]. The molecular mechanisms controlling the tachyzoite-bradyzoite-stage conversion in *N. caninum* are not well understood, as reliable in vitro models to study bradyzoite development have not been set up. In vitro stage conversion has been initiated by specific stress conditions such as pH changes and nitric oxide exposure [13,14,15], but the efficiency of these methods is limited [16]. 

**Figure 1:**
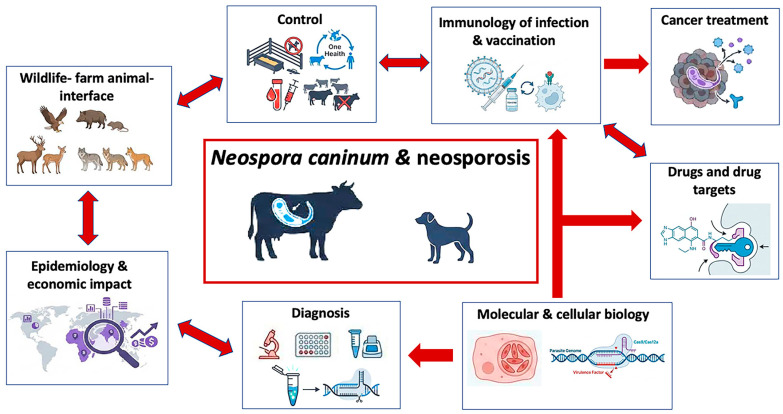
Integrated research framework for *Neospora caninum* and neosporosis organized into eight thematic sections, partially interlinked as indicated by the red arrows. These include diagnosis (traditional and more recently developed methods), epidemiology and economic impact, the interface of wildlife and farm animals, control measures to limit infection and transmission of the disease, most notably in cattle, molecular and cell biological studies that identify and characterize virulence factors and potential vaccine and drug targets, and exploiting *N. caninum* as a cancer immunotherapy platform. Arrows indicate which research areas have impacted each other. Images were generated through NotebookLM.

The clinical manifestations of neosporosis vary, depending on the host species. In cattle, the primary impact is on reproductive health. Abortion mostly occurs in the second trimester of pregnancy, but neosporosis can also lead to birth of weak offspring or persistently infected calves devoid of any clinical signs. However, in subsequent pregnancies, these healthy offspring can transmit the parasite to the next generation, which represents a severe complication for the introduction of control measures [17]. Thus, early and accurate diagnosis is crucial for the effective management. In dogs, *N. caninum* infection can cause severe neuromuscular disease [1]. Neurological manifestations are often multifocal with predominantly cerebello-vestibular signs. The prognosis is generally poor, with complete clinical improvement in only about 6% of cases and a relapse rate of >25% [18]. Clinical presentations vary with age. In dogs under six months, manifestations primarily involve the peripheral nervous system with more generalized neuromuscular disorders including decreased spinal reflexes, tetraparesis, progressive weakness, muscle loss, and myalgia. In dogs over one-year old, the central nervous system appears affected to a higher degree, with cerebellar ataxia, head tremors, vestibular syndrome, and facial nerve deficits. Additional manifestations can include mega-esophagus, hyperthermia, and hepatomegaly [19]. In puppies, particularly those infected transplacentally, the infection can be particularly severe and often leads to death [20]. *N. caninum* imposes substantial economic burden most notably on the global cattle industry. Thus, vaccine development remains a primary research focus. Overall, traditional vaccine approaches have shown limited efficacy, but significant advances in reverse vaccinology, incorporating modern in silico methods and advanced genomic analyses, offer new opportunities for identifying promising vaccine candidates [7,21]. The complex nature of *N. caninum* transmission necessitates a One Health approach, recognizing the interconnections between livestock, wildlife, and human activities. This approach requires sustained international collaboration and coordinated surveillance, while considering ecological contexts and environmental factors [22]. To visualize the interconnected research domains and translational pathways discussed in this review, a schematic framework integrating diagnosis, epidemiology, molecular and immunological mechanisms, intervention strategies, and future directions is presented in Figure 1.

In this review, we summarize information on key advances in the field over the last 10 years and relate them to important findings published earlier.

## 2. Diagnosis of *N. caninum* Infection

The diagnosis of *N. caninum* infection relies on indirect serological detection of infection and direct demonstration of the parasite via molecular tools and immuno-histopathology. Serological techniques such as enzyme-linked immunosorbent assay (ELISA) and indirect fluorescent antibody test (IFAT) have been widely used in different animal species [6,23]. ELISA, using crude antigens or subcellular fractions, offers high throughput for large-scale screening, while IFAT on intact tachyzoites provides high specificity. However, ELISA may present cross-reactivity issues, and IFAT is labor-intensive. In addition, a plethora of different PCR-based techniques were established. A brief overview of the diagnostic laboratory methods that have been developed since the discovery of *N. caninum* is shown in Table 1.

More recently, improvements in ELISA have been achieved through the development of chimeric antigens, such as a recombinant antigen based on the two surface antigens NcSRS2 and NcSAG1 and the dense granule antigen NcGRA7 [34], achieving high sensitivity (86.7%) and specificity (96.1%) in detecting *N. caninum* antibodies in cattle. Udonsom et al. [35] evaluated the immunodiagnostic performances of specific recombinant proteins including NcPrx2, NcMIC4, and NcSAG1, with NcSAG1 and NcMIC4 demonstrating particular promise as reliable diagnostic markers. Fereig et al. [36] validated a NcSAG1-based immunochromatographic test (ICT) for the detection of *N. caninum* infection in cattle, which demonstrated high sensitivity (84.2%) and specificity (93.5%). This ICT is a rapid tool for on-site diagnosis, representing a significant advancement in field-applicable diagnostics for neosporosis control. To complement traditional diagnostic approaches and to identify active infections, quantification of pro-inflammatory cytokines such as IL-12 and IFN-γ can aid in distinguishing chronic and active infections [37], which is relevant given that *N. caninum* can establish persistent infections with varying levels of immune response at different stages.

Histopathological examination, particularly immunohistochemistry (IHC), remains the gold standard for definitive diagnosis. In cases of abortion, examination of both placental and fetal tissues is crucial. Advances in immunodiagnostics have improved the ability to differentiate between *N. caninum* and *T. gondii* in tissue samples. Lepore et al. [38] developed species-specific polyclonal antibodies targeting recombinant proteins (rNcSRS2 and rTgSRS2) that achieved 90.1% agreement between IHC and PCR testing. This advancement addresses a critical challenge in diagnostic specificity, particularly in abortion cases. While IHC is highly useful and informative, the quality and reliability of results depend significantly on several factors including infection stage, tissue preservation methods, and timing of sample collection. The characterization of stage-specific immunodominant antigens has further enhanced our understanding of diagnostic targets. For instance, evaluation of monoclonal and polyclonal antibodies against different parasitic stages demonstrated that antibodies directed against NcSAG1 specifically recognized *N. caninum* tachyzoites, while antibodies directed against the bradyzoite antigen NcSAG4 displayed broader cross-reactivity among parasite stages [39]. These findings have important implications for the development of stage-specific diagnostic tools and highlight the need to consider developmental stages when designing diagnostic tests.

*N. caninum* diagnosis has significantly benefited from advances in molecular techniques, providing unparalleled sensitivity and specificity. Quantitative PCR (qPCR) targeting the Nc5 gene enables detection of as few as 5 copies/µL in complex samples [40]. Building on qPCR technology, a high-resolution melting (HRM) assay developed by Gouvias et al. [41] enabled simultaneous detection of ten abortifacient pathogens including *N. caninum* in one sample, providing a cost-effective diagnostic solution for multiple pathogen screening. Optimized multiplexed qPCR assays allow simultaneous detection of *N. caninum* and *T. gondii* with a detection limit of less than a single tachyzoite [42]. Droplet digital PCR (dPCR) could be a further improvement to enhance diagnostic precision. In the case of *T. gondii*, dPCR achieved absolute quantification, with strong concordance (96.7%) with qPCR results and detection limits as low as 0.07 copies/µL [43], highlighting the potential application of dPCR also for *N. caninum* diagnostics. Emerging platforms, such as Loop-Mediated Isothermal Amplification (LAMP) and CRISPR-based systems, continue to push the boundaries of diagnostic innovation. LAMP demonstrated field applicability with sensitivity and specificity exceeding 90% [44,45]. Further validation of LAMP technology comes from Liu et al. [46], who developed a colorimetric detection method that enables reliable visual identification of *N. caninum* DNA without specialized equipment. In addition, CRISPR-based detection platforms have shown increasing promise, such as a recombinase polymerase amplification (RPA)-CRISPR/Cas12a-based detection using fluorescent readouts [47].

Numerous commercial diagnostic kits for serological and molecular detection of *N. caninum* infection are available on the market. However, bridging the gap between the laboratory- and field-based diagnosis by the integration of CRISPR and especially LAMP into portable kits could enormously advance sensitive and timely diagnosis. Importantly, each diagnostic technique exhibits its distinct advantages for specific applications. PCR-based molecular methods, while technically demanding, provide highly sensitive and specific detection of *N. caninum* [48], with advanced multiplexed qPCR systems achieving detection limits of less than one tachyzoite [42]. IFAT serves as a reference method, though its labor-intensive nature limits large-scale applications and subjective interpretation can be a problem [35]. ELISA provides a practical tool for herd-level screening despite varied kit agreement [49]. Emerging platforms such as RPA-CRISPR-based detection show high sensitivity with detection limits of one parasite/mL using fluorescent systems [47], while LAMP, not requiring extensive infrastructure, offers field applicability [45].

## 3. Distribution and Transmission of Neosporosis in Farm Animals

Earlier studies had shown that *N. caninum* infection in cattle is distributed worldwide with prevalence rates ranging from 10% to 60% (reviewed in [6]). Intensive farming systems generally showed a higher risk of infections with prevalence rates appearing to be influenced by management practices and animal husbandry.

In Europe, varying levels of *N. caninum* prevalence have been recorded in the last 5 years. In Switzerland, an overall low seroprevalence of 4.2% was observed in female cattle, with 16.2% of farms having at least one seropositive animal [50]. In Portugal, the animal-level seroprevalence was 17.2% and the herd-level seroprevalence was 68.6%, with notable differences between production systems—dairy farms showed a higher prevalence of 26.8%, compared to beef farms (14.4%) [51]. A large-scale study carried out on dairy cows in Northern Italy discovered significant regional variations in *N. caninum* prevalence. Analysis of bulk tank milk from 586 dairy herds showed an overall seroprevalence of 30.7%, with a higher prevalence in small−medium farms and older animals. Environmental factors such as temperature and altitude, as well as land use patterns, significantly influenced prevalence rates in different areas [52]. Studies on *Neospora* seroprevalences in bulk milk were also carried out in Canada, where a seasonal study in Alberta showed fluctuating prevalence rates throughout the year, ranging from 7.4% to 18.2% [53]. In Colombia, an overall seroprevalence of 20.6% was reported, with notably higher rates in beef herds compared to dairy herds [54]. In China, comprehensive meta-analyses were carried out. For instance, a nationwide review found an overall seroprevalence of 12.2% (20.9% in Southern China, 9.4% in Northwest China) [55]. Another meta-analysis examining the results obtained from 33,945 cattle from 51 studies across mainland China found an overall prevalence of 13.69% [56]. At the provincial level, significant variations were observed, with particularly high rates in Hebei Province where 37.34% of dairy cows tested positive [57], while lower rates were found in Hunan Province with 2.1% seroprevalence in beef cattle [58]. In Central India, a study of 576 cattle revealed a seroprevalence of 24.8%, with risk factors including age, movement of dogs on farms, drinking pond water, and history of abortion [59]. The first molecular study carried out in Bangladesh [60] identified *N. caninum* in aborted fetuses by nested PCR, with infection rates of 16.0% in cattle, 14.8% in sheep, and 11.8% in goats. Contrasting patterns between regions were also found on the African continent. In Egypt, two studies showed consistently high seroprevalence rates: 28.9% in Northern governorates [61] and 24.6% in the Beheira region [62]. In contrast, South Africa demonstrated much lower rates with an overall seroprevalence of 2.3%, although significant regional variations ranging from 7.5% in KwaZulu-Natal to 0.1% in Western Cape were observed [63]. In Somalia, a comprehensive survey revealed a seroprevalence of 3.6% in ruminants, with cattle showing the highest prevalence (6.2%), followed by goats and sheep (both 2.2%) [64]. An unpublished serological survey was conducted in the Biskra and Aures regions of Algeria. Of 64 serum samples obtained from female cattle, 8 were found positive (Debache et al., unpublished findings). Although serological screening can be informative, the antibody titers in persistently infected animals can fluctuate considerably. A recent study from De Oliveira et al. [65] demonstrated significant alterations in *N. caninum* antibody titres throughout pregnancy in naturally infected crossbred cows, with notable increases in serological titration per trimester. These findings suggest that pregnancy influences antibody levels and that serological testing from the sixth month of gestation onwards may reduce false negative results. In South America, bovine neosporosis represents a serious veterinary and economic problem. Neosporosis accounts for approximately 9% of bovine abortions in Buenos Aires Province, and epidemiological studies across Argentina revealed a high distribution of *Neospora* infections not only in dairy but also in beef cattle, with seroprevalence rates of 16.6–88.8% and 0–73%, respectively [66]. One study carried out in Brazil identified *N. caninum* as the primary cause of bovine abortion in 53.8% of cases, with PCR confirmation in 71.4% of positive samples [67]. Further epidemiological studies in Brazil’s southern region have revealed important environmental risk factors, with the presence of dogs being the most important one. In Santa Catarina State, Remor-Sebolt et al. [68] reported a 4.2% seroprevalence in canine populations, with housing conditions and outdoor exposure significantly influencing infection rates. A study of free-roaming dogs in Ecuador found a seroprevalence of 6.8%, with no significant differences between urban and rural areas, highlighting the widespread distribution of *N. caninum* in canine populations across diverse environments [69]. In contrast, lower seroprevalences in dogs and cats were measured in urban settings in Poland, with rates of 1.0% in dogs and 3.3% in cats [70]. This highlights the importance of environmental factors in *N. caninum* transmission, particularly in areas where dogs have unrestricted access to outdoor spaces and can thus influence the transmission dynamics.

*N. caninum* is transmitted either vertically from the dam to the fetus or horizontally through the ingestion of oocysts containing sporozoites or infected meat containing tissue cysts and bradyzoites. Vertical transmission represents the predominant route in both domestic and wild hosts and remains the most critical barrier to effective control. In one study on Iranian dairy cattle, vertical transmission resulted in 13.6% infection rates among offspring of seropositive dams, with 94.1% of aborted fetuses being *N. caninum*-positive [11]. Lagomarsino et al. [71] reported horizontal transmission rates of *N. caninum* reaching 22.7% in dairy cattle in an endemic region in Argentina. In South American deer, transmission rates of >80% were recorded [72], and in goats, rates reached up to 100% across successive generations [65]. Studies with wild ungulates in Northern Italy demonstrated an efficiency of congenital transmission reaching 87.5% [73], highlighting the significance of this pathway in maintaining parasite populations.

Horizontal transmission through ingestion of sporulated oocysts or the oral uptake of meat infected with *N. caninum* tissue cysts is influenced by environmental factors and host diversity. In Brazilian cattle, the access of dogs to pastures and improper disposal of fetal remains were key risk factors [74], and environmental factors such as altitude, precipitation, and temperature significantly affected *N. caninum* seropositivity rates [75]. Management practices focusing on preventing definitive host access to cattle feed and water sources remain critical for controlling horizontal transmission. The complexity of transmission dynamics, involving both domestic and wildlife cycles, necessitates integrated approaches to disease control. In this respect, a 2023 meta-analysis revealed a global seroprevalence of 5% in rodents, confirming their potential role as reservoir hosts [76].

The worldwide prevalence of neosporosis has a considerable economic impact. An earlier systematic review by Reichel et al. [77] estimated global losses exceeding US $1.298 billion per annum, with nearly two-thirds of the losses (US $842.9 million) incurred by the dairy industry. Ribeiro et al. [78] reported that *N. caninum*-infected cattle were 2.66 times more likely to experience abortion, highlighting the direct link between infection rates and economic losses. A more recent report from Turkey [79] estimated costs associated with *Neospora* infection of approximately 710 US$ per dairy cow. In Australia, studies have consistently estimated annual losses at AU$110 million [80,81], while in Argentina the annual economic impact amounts up to US$33 million in the dairy cattle and 12 million in the beef cattle industry [66]. Financial losses through neosporosis are particularly pronounced in regions with intensive cattle production systems, and respective assessments are not straightforward, due to the differences in surveillance capabilities across regions. Despite the importance of livestock production for food security and livelihoods in many communities, comprehensive surveillance data for abortifacient pathogens including *N. caninum* remain limited in many parts of Africa and Asia [82]. This gap in surveillance suggests that the true global economic impact may be substantially underestimated, highlighting the need for improved monitoring and control strategies.

## 4. The Farm Animal−Wildlife Animal Interface and Control

Due to its implications for livestock health, the role of wildlife as reservoirs for *N. caninum* has garnered increasing attention. The presence of *N. caninum* was reported in migratory birds, underscoring their potential as long-distance vectors contributing to the geographic spread of the parasite [73]. Other studies identified elevated transmission risks in regions where domestic animals interact closely with wildlife [83]. In addition, wild ungulates acting as intermediate hosts were suggested to contribute to the presence of *N. caninum* in livestock [84]. Additional wildlife reservoirs were identified by Haydett et al. [85] and Zanet et al. [73] in wild pigs and birds of prey. In a long-term study lasting 18 years, the seroprevalence of *N. caninum* in wild rabbits was shown to be >6%, with significant seasonal variations and peak rates during spring, most likely related to prolonged oocyst survival [86]. Huaman et al. [81] detected a 3.7% seroprevalence in wild deer across Southeastern Australia, and Baldini et al. [72] demonstrated an 81.25% vertical transmission rate of *N. caninum* in South American deer. Thus, control approaches for *N. caninum* should also take into account the contribution of the sylvatic cycle.

Spatial analyses in endemic regions of the Amazonas in Brazil have revealed significant heterogeneity in parasite distribution, with seroprevalences ranging from 2.2% to 69.2% [87]. Similarly, epidemiological studies in Argentina revealed seroprevalence rates ranging from 16.6% to 88.8% in dairy cattle and 0% to 73% in beef cattle, demonstrating dramatic regional differences. Thus, it is important to integrate genetic, environmental, and management factors into tailored intervention strategies to mitigate the diverse impacts of *N. caninum* [66]. Key risk factors for neosporosis were identified, including dairy farming systems, mixed land use patterns, water source management, and the presence of domestic dogs, with the latter as a continuous source of environmental contamination with oocysts [78,87]. Another factor that influences the epidemiology of neosporosis is genetic variability. A study on *N. caninum* isolates from aborted bovine fetuses carried out in Northern Italy showed that spatial distance between sampling sites can be linked to genetic variation [88]. Thus, localized adaptation can potentially lead to genetic divergence. In the Amazonian region of Brazil, environmental factors such as high humidity, extensive floodplains, and dense livestock−wildlife interfaces significantly impact parasite transmission [87].

Host−pathogen interactions further complicate integrated control strategies. Holstein cattle show increased susceptibility to *N. caninum*-induced abortion compared to beef breeds [89], while embryo transfer studies demonstrate that donor seropositivity can affect embryo quality without direct transmission [90]. In Argentina, selective breeding strategies and embryo transfer have demonstrated efficacy in reducing vertical transmission and seroprevalence in dairy herds, providing a promising avenue for mitigating reproductive losses [66]. Experimental infections of Texel sheep with *N. caninum* at different stages of gestation demonstrated vertical transmission of the parasite to the fetus, but absence of abortions and other clinical signs suggested that Texel sheep may potentially have resistance to *N. caninum*-induced abortion [91]. These findings suggest that genetic and reproductive management strategies must be considered alongside broader epidemiological interventions.

## 5. Molecular and Cellular Biology of *N. caninum*

Significant progress in molecular techniques has revolutionized our understanding of *N. caninum* through multiple technological breakthroughs. High-throughput sequencing analysis has already earlier revealed novel insights into the genome of *N. caninum*, such as the presence of an expanded repertoire of SAG1-related proteins compared to *T. gondii* [92]. More recent studies have employed high-throughput RNA sequencing to study microRNAs (miRNAs) involved in the regulation of gene expression in parasites and host cells. For instance, Liu et al. [93] identified 300 miRNAs in *N. caninum* tachyzoites, and bioinformatics analyses showed that 10 were conserved among metazoan miRNA families, while 290 were novel miRNAs. In another bioinformatics study, Das et al. [94] performed a homology search on 336 non-redundant Expressed Sequence Tags (ESTs) of the *N. caninum* genome to identify conserved miRNAs. A total of 1041 mature miRNAs of reference organisms were employed; one putative miRNA “nca-miR-9388-5p” of 19 nucleotides was identified, and 16 potential target genes associated with different protozoal physiological functions were identified. This work has been complemented on the host cell side by temporal transcriptomic analyses of miRNA expression in caprine endometrial epithelial cells infected with *N. caninum* at 24 h and 48 h post-infection, identifying numerous up- and downregulated miRNAs at both time points. Bioinformatically predicted targets are genes involved in host immune responses, metabolism, and multiple signaling pathways. One upregulated chi-miR-146a was found to promote tachyzoite proliferation [95].

A pivotal breakthrough in genetic manipulation tools came with the implementation of CRISPR technology. Arranz-Solís et al. [96] established an efficient gene disruption system using CRISPR/Cas9 plasmids earlier developed for *T. gondii*, successfully targeting GFP in a reporter strain and disrupting NcGRA7 through pyrimethamine-resistance cassette (*mdhfr-ts)* insertion. However, it was recognized that CRISPR/Cas9 mutagenesis is not free of off-target effects that can lead to integration of multiple *mdhfr-ts* copies into other sites of the genome. Thus, TaqMan-quantitative PCR assays were developed that allowed determination of the copy numbers of the integrated selectable markers in CRISPR/Cas9-generated *N. caninum* KO strains [97]. To prevent multiple insertions, another highly efficient genetic manipulation system was established by Mineo et al. [98] through disrupting the *ku80* gene in the *N. caninum*-Liverpool reference strain. This modification led to improved homologous recombination efficiency and enabled precise gene targeting with shorter homology regions, bringing the manipulation capabilities closer to those available for *T. gondii*.

The work by Arranz-Solis et al. [96] was closely followed by Nishikawa et al. [99] who reported that NcGRA7 KO strains exhibit a reduced virulence and that this protein is involved in modulation of the immune response [99]. The same was shown in the pregnant neosporosis mouse model [100]. In addition, NcGRA7 was shown to interact with cellular immune factors in mice, such as mediating the aggregation of IRGa6—an interferon-inducible GTPase—at the PVM, which affects the pathogenicity of *N. caninum* [101]. However, cross-species translation of results such as from mice to farm animals should be done with caution. The basic features of murine and bovine immunity, including transplacental passage of immunoglobulins, Th1- and Th2-immunity, the composition of the T cell repertoire, and the functionality of interferon-induced GTPases, show clear differences [7].

Other GRA proteins were characterized by generating CRISPR KO strains such as NcGRA17 [102], NcGRA6 [103], and NcGRA2 [104], demonstrating that these proteins were important for parasite virulence. Biotinylation revealed interaction partners with NcGRA17, named NcGRA23 and NcGRA11, but the corresponding CRISPR KO strains did not show any impairment. Three novel GRA proteins, NcGRA27, NcGRA61, and NcGRA85, were identified by the proximity-dependent biotin identification (BioID) technique [105]. Deletion of the NcGRA27 gene reduced the in vitro replication and the pathogenicity of *N. caninum* tachyzoites in mice, while deletion of NcGRA61 and NcGRA85 had no impact on virulence [105]. CRISPR/Cas9 was also employed to clarify the roles of two rhoptry proteins as virulence factors, namely NcROP40 and NcROP2 in *N. caninum* development and host−parasite interactions. NcROP40 KO strains exhibited a modestly reduced virulence compared to wild-type parasites when assessed in the pregnant mouse model [100], while NcROP2 KO tachyzoites exhibited more impaired virulence in the pregnant neosporosis mouse model, were less susceptible to IFN-γ-mediated inhibition and were more readily converted to the semi-dormant bradyzoite stage compared to the wild type [106]. The transcription factor NcAP2XII-4, whose expression was previously shown to be altered upon deletion of the gene coding for NcROP5, was also functionally characterized by employing CRISPR/Cas9 [107]. CRISPR/Cas9-mediated deletion of NcAP2XII-4 resulted in parasites that were not able to undergo egress from the parasitophorous vacuole and could not form plaques, and subsequent investigations confirmed that NcAP2XX-4 regulated ROP5 transcription by binding to its promoter. Other proteins whose roles as virulence factors were confirmed are the major surface antigen NcSAG1 as a major determinant mediating pathology in pregnant and non-pregnant mice [108], the virulence factors NcPuf [109], and the c-myc-proto-oncogene regulatory protein NcMyr1 [110].

Besides these molecular advances, classical and modern microscopy approaches were applied to characterize endodyogeny in *N. caninum* tachyzoites [111]. Expansion microscopy and regular confocal microscopy were used to study the dynamics of the cell assembly and nuclear division, describing centrosome, centriole, and apicoplast dynamics, during the cell cycle. In addition, the implementation of three-dimensional culture systems has opened new avenues for studying host−pathogen interactions, especially in apicomplexan parasites related to *N. caninum* such as *Plasmodium*, *Cryptosporidium*, *Eimeria*, and *Toxoplasma* [112,113]. Such cultures, including organoids and microfluidic devices, provide more physiologically relevant contexts for studying parasite life cycles and the implementation of advanced molecular techniques.

## 6. Immunological Control of Infection and Vaccination

The host immune response against *N. caninum* infection has a profound influence on the outcome of pregnancy and fetal survival. In general, as for many other intracellular pathogens, a Th1-biased immune response characterized by pro-inflammatory cytokine expression mediates protection against *N. caninum* infection. However, it has been shown in murine models that pregnancy outcome can be impaired due to excessive inflammatory responses [12,114], and the same could be true for bovine hosts [115]. A further factor complicating the situation is the fact that different *N. caninum* isolates were shown to differ considerably in virulence. Differences in terms of innate and adaptive immune responses were observed in a study on placentomes of cattle experimentally infected with a highly virulent (Nc-Spain7) isolate and a low-virulence (Nc-Spain 1H) isolate [116]. Infection with the low-virulence isolate resulted in the upregulated expression of pathogen recognition receptors, chemokines, and pro-inflammatory cytokines that mediate control, as well as other mechanisms implicated in the maintenance of extracellular matrix integrity, resulting in ensuring fetal survival. In contrast, these responses were impaired in placentomes of cattle infected with the more virulent Nc-Spain7 isolate during the first 10 days post-infection (10 dpi). Subsequently (20 dpi), a predominantly pro-inflammatory Th1-based response and increased leucocyte infiltration were observed, and fetal death was associated with higher expression levels of IL-8, TNF-α, iNOS, and SERP-1 genes, and lower expression of the metalloproteases and their inhibitors, compared to placentomes from animals carrying viable fetuses [116]. Understanding these immunological mechanisms is essential for developing effective control strategies based on vaccines, not only in cattle but also in sheep and goats [117].

Early efforts in vaccine development, carried out in murine models and to a lesser extent also in small and large ruminant models, included vaccines composed of *N. caninum* tachyzoite antigen extracts and/or subcellular fractions and those composed of recombinant antigens as monovalent and chimeric formulations, as well as polyvalent recombinant vaccine candidates expressed in different expression systems and formulated with different adjuvants (reviewed in [7]). Table 2 summarizes a range of studies which were done in the actual target hosts, namely one study in sheep [118] and others in cattle, employing either parasite extracts/subunit vaccines or live vaccines (attenuated *N. caninum* isolates). With one exception [118], attenuated live vaccines were those that provided meaningful protection against abortion and/or vertical transmission, while subunit vaccines showed lower efficacy [7].

Most vaccine candidates studied to date are immunogenic surface, microneme, and rhoptry of dense granule proteins functionally involved in host cell adhesion/invasion. Parasite proteins that elicit inflammatory responses and are responsible for the immunopathology that leads to abortion have also been considered as vaccine candidates. Of note, a dual-antigen vaccine combining recombinant *N. caninum* cyclophilin (NcCyP) and profilin (NcPro), both important mediators of inflammatory responses, has shown significant protection in sheep, with 69.2% of vaccinated ewes giving birth to viable lambs compared to complete abortion in control animals [118]. More recently, Mendoza-Morales et al. [133] introduced a recombinant subunit vaccine with a dual Differentiation of Infected from Vaccinated Animals (DIVA)-like capability, which comprises recombinant NcSAG1 and the carrier/adjuvant heat shock protein 81.2 from *Arabidopsis thaliana* (rAtHsp81.2, a known B-cell mitogen). The vaccine formulation has been demonstrated earlier to be safe and efficacious in the murine neosporosis model and allowed differentiation between vaccinated and infected animals [134]. In cattle, this formulation stimulated a broad and potent humoral and cellular immune response, characterized by an IgG1/IgG2 isotype profile and IFN-γ secretion, and also allowed differentiation between vaccinated and infected heifers by two different DIVA compliant test approaches [133]. Studies on the efficacy of this vaccine approach are still pending, but using this strategy will allow more precise disease monitoring, making it a critical step forward in controlling *N. caninum* in cattle.

Although in earlier studies the most promising vaccine results were obtained with live-attenuated parasites in both murine models [135,136,137,138] and cattle [127,128,129], safety concerns, production costs, and the anticipated non-stability of live vaccines blocked their further development into commercial products [7]. As an alternative, an approach that utilizes the attenuated *Listeria monocytogenes* vaccine vector Lm3Dx devoid of three important virulence genes but expresses one or more *N. caninum* antigens has been introduced. The safety and outstanding efficacy of this Lm3Dx-based *Neospora* vaccine vector has been demonstrated in the pregnant neosporosis mouse model [139,140,141].

Additionally, immunoinformatic approaches have been followed for the virtual to design and evaluation of vaccine candidates, predicting a number of important features such as antigenicity, solubility, potential allergenic domains, posttranslational modifications, transmembrane domains and signal peptides, secondary and tertiary structures, and linear and conformational B-cell epitopes, and potential MHC class I and II presentation and T cell cytotoxicity. Using this approach, Shams et al. [142] predicted a multiepitope vaccine candidate incorporating epitopes from six key proteins (SRS2, MIC3, MIC6, GRA1, IMP-1, and profilin). In addition, bioinformatic evaluation of the NcSRS2 protein identified immunogenic epitopes capable of inducing dual immune responses, with high antigenicity and no allergenicity, positioning it as a strong candidate for next-generation vaccines [143].

Progress has also been made in the development of delivery methods and formulations. Yao et al. [144] highlighted the importance of adjuvant combinations for preventing high-incidence diseases in bovines, such as the addition of TLR2 and TLR9 as adjuvants for recombinant NcPro to achieve higher levels of IFN-γ and to ensure a prolonged recall B-cell response in cattle compared to other adjuvants [124]. Others have used di-palmitoyl phosphatidyl glycerol-loaded nanoparticles (DGNP) loaded with *N. caninum* extract and *N. caninum* glycosylphosphatidylinositol (GPI) and found an adjuvant effect in murine bone marrow-derived dendritic cells with higher levels of interleukin (IL)-1β, IL-6, IL-12p40, and IL-10, and decreased expression of major histocompatibility complex (MHC) molecules. GPI also modulated the responses of bovine peripheral blood mononuclear cells (PBMCs) by increasing the production of IFN-γ and by decreasing the expression of MHC molecules [145], suggesting that the GPI adjuvant effect should be exploited for vaccination. In addition, recent developments of more advanced DNA vaccine platforms [146], self-assembling protein nanoparticles (SAPNs), and virus-like particles (VLPs) with demonstrated abilities to enhance antigen uptake, B-cell activation, and lymph node trafficking [147] await further exploitation in the *Neospora* vaccine field.

Subunit vaccine candidates could also be incorporated into mRNA vaccines. As reviewed in [7], mRNA can overcome mis-folding during in vitro expression of recombinant proteins, and mRNAs, as opposed to DNA vaccines, are considered to have very little safety issues as they retain a cytoplasmic localization, thus minimizing the risk of gene recombination and conversion into malignant cells. Messenger RNA vaccines are designed relatively easily, thus incurring little costs of production and time requirements. The major caveat is their short intracellular half-life and the rapid degradation during storage [7]. However, variability in host immune responses remains a significant barrier, necessitating further standardization and optimization.

## 7. Recent Advances in Therapeutic Strategies

Traditional therapeutic approaches against *N. caninum* have relied on folate biosynthesis inhibitors, primarily sulfadiazine and pyrimethamine, or alternatively a combination of trimethoprim, another folic acid antagonist, and sulfamethoxazole. These compounds, while useful for managing clinical neosporosis in dogs, show limited efficacy in cattle, particularly due to their potential adverse effects in pregnancy and their minimal impact on tissue cysts [148,149]. Other repurposed drugs have been investigated but in many cases have not been applied clinically in cattle such as the protein synthesis inhibitor clindamycin, the ionophore antibiotic monensin, the triazinetrione derivative toltrazuril, the cytochrome bc1 inhibitors decoquinate, buparvaquone and endochin-like quinolones (ELQs), a number of anti-malarials such as artemisinin and its derivatives and mefloquine, and the alkyl phospholipid miltefosine (for review see [7,150]). Systematic screening efforts of open-source libraries such the Medicines for Malaria Venture (MMV) Malaria Box and the MMV Pathogen Box employing transgenic parasites that express bacterial beta-galactosidase have significantly expanded the arsenal or repurposed drugs [151,152]. More recently, CRISPR/Cas9 was used to generate transgenic tachyzoites that allow high-throughput in vitro drug screening [153], leading to the discovery of TAK-632, a selective pan-rapidly accelerated fibrosarcoma (pan-RAF) kinase inhibitor employed in cancer. In experimentally infected mice, TAK-632 attenuated the virulence of *N. caninum* and significantly reduced the parasite burden in the brain.

Additionally, Harada et al. [154] developed a novel high-standard chemiluminescent assay targeting nucleoside triphosphate hydrolase (NTPase), leading to the identification of 19 synthetic compounds and six marine bacterial extracts with inhibitory activity, providing potential candidates for anti-parasitic drug development. This screening platform was further refined by Kurata et al. [155], who implemented robotic automation to enhance throughput and precision, establishing a more efficient drug discovery pipeline for both *N. caninum* and *T. gondii*. A comprehensive evaluation of antimalarial compounds against *N. caninum* revealed distinct efficacy profiles [156]. Atovaquone emerged as the most potent compound (IC50 = 8 nM), comparable to its activity against *T. gondii* and *Plasmodium.* Tetracycline showed significant efficacy with an IC50 of 19.6 μM, while primaquine and quinine demonstrated very low activity (IC50 = 44.4 μM and 56.6 μM, respectively). Notably, chloroquine required concentrations above 100 μM for inhibitory effects. The study also revealed important differences in drug susceptibility patterns among apicomplexan parasites, suggesting species-specific therapeutic targets [156].

Another example of a repurposed drug is niclosamide, a compound that was developed for clinical use in tapeworm infections and blocks glucose uptake. Niclosamide was shown to act against *N. caninum* through a novel mechanism involving the NLRP3 inflammasome activation in mice. Niclosamide enhanced macrophage-mediated parasite clearance but also exhibited direct antiparasitic activity, significantly reducing the parasite burden in tissues and improving survival rates in murine models. Notably, niclosamide treatment affected the mitochondrial membrane potential and ATP production in *N. caninum* tachyzoites, suggesting multiple mechanisms of action [46].

The efficacy of natural products such as a cyclic natural compound isolated from *Tetragonisca angustula* honey was demonstrated, exerting a 40–56% reduction in tachyzoite numbers and a dose-dependent inhibition of parasite proliferation up to 50%, all without toxicity to host cells [157]. Another natural compound, *Inonotus obliquus* polysaccharide (IOP) was identified by Tang et al. [158]. IOP treatment at 2 mg/10 g provided protection against *N. caninum* infection in mice, reducing parasite burden in multiple organs and modulating immune responses through regulation of immunoglobulin levels (IgG1, IgG2a) and cytokine production (IL-12, TNF-α). Additionally, IOP treatment showed no toxicity in vitro or in vivo, while effectively balancing hormonal responses, suggesting its potential as a safe therapeutic option [158]. Cordycepin, a nucleoside antibiotic derived from Chinese medicine *Cordyceps militaries*, was shown to exhibit profound activity against *N. caninum* in vitro and in vivo. Upon experimental infection, mice treated with cordycepin showed reduced clinical symptoms, increased food intake and significantly increased body weight [159].

More targeted therapies have been achieved with Bumped Kinase Inhibitors (BKIs). These compounds target two apicomplexan kinases, namely calcium-dependent protein kinase 1 (CDPK1) that is involved in signaling events that regulate host cell invasion and egress [160], as well as a specialized mitogen-activated protein kinase (MAPKL1) that localizes to the centrosome where it prevents overduplication and is crucial for proper centrosome function during asexual replication and cell division [161]. The latest generation BKIs, namely BKI-1748 and BKI-1708, efficiently impaired *N. caninum* tachyzoite proliferation (IC50 < 500 nM) and effectively reduced vertical transmission in experimentally infected mice [162,163]. Interestingly, BKIs do not kill the parasites in vitro but induce the formation of multinucleated complexes consisting of newly formed zoites that are blocked in the final stages of cell division and remain stuck within the host cell cytoplasm. This affects the humoral and cellular immune response as shown in experimentally infected mice treated with another BKI-family member, BKI-1294 [138], as well as with BKI-1748 and BKI-1708 [162,163]. By applying affinity chromatography, BKI-1748 was shown to bind not only to NcCDPK1 but also to *N. caninum* proteins and enzymes that are crucially involved in RNA- and DNA-binding and -modification processes, most notably splicing factors, suggesting that other targets besides the activity against these two kinases are involved [164].

The field has progressed through combination approaches and drug delivery innovations. Anghel et al. [165] studied the efficacy of ELQs that were originally developed for the treatment of toxoplasmosis, with one derivative, ELQ-334, achieving a 50% reduction of vertical transmission in pregnant mouse models. A treatment with a combined ELQ-334 plus BKI-1748 treatment achieved a synergistic effect in vitro and resulted in complete inhibition of vertical transmission in the mouse model [166]. Efforts on drug delivery options are underway to improve bioavailability, pharmacokinetic properties, and targeted action of compounds. This could be achieved by formulating compounds in solid dispersions, microparticles, polymeric micelles, nanosuspensions, lipid-based nanocarriers or liposomes, and other suitable agents.

Modulating the host immune response to combat *N. caninum* infection offers another promising therapeutic pathway. High and low virulence isolates of *N. caninum* induce virulence-dependent pro-inflammatory responses in bovine macrophages via the NF-κB signaling pathway [167]. These differences provide a foundation for therapeutic interventions aimed at enhancing protective immunity while reducing parasite evasion mechanisms. Targeting host signaling pathways offers further opportunities. Bhandage et al. [168] revealed that *N. caninum* activates GABAergic signaling in mononuclear phagocytes, enhancing their motility and facilitating parasite dissemination. Therapeutic strategies that disrupt GABA receptor or calcium signaling could limit parasite spread. Similarly, Mota et al. [169] identified the p38 MAPK pathway as a critical mechanism of immune evasion, with its inhibition leading to increased cytokine production and improved host survival. Additional research has focused on host-based strategies.

## 8. *N. caninum* as a Therapeutic Agent for Cancer Treatments

Most interestingly, within the last few years, *N. caninum* has switched its role from being a therapeutic problem to becoming a therapeutic agent, most notably in cancer treatment. Lantier et al. [170] exploited the following facts: (i) tumor growth induces an immune-modulated environment; and (ii) infection with an intracellular pathogen can potentially twist this modulated, or partially downregulated, immunity into eliciting an inflammatory response that could also affect tumor cells. Injection of *N. caninum* tachyzoites into mice inoculated with thymoma EG7 or human Merkel cell carcinoma led to severe impairment or even eradication of the tumor mass. This effect was based not only on direct lysis of tumor cells, but also on the reactivation of immunosuppressed cells and the development of a protective anti-tumor response dependent on natural killer (NK) cells, CD8-T cells, and interferon (IFN)-γ secretion into the tumor microenvironment. A transgenic *N. caninum* strain expressing and secreting human interleukin (IL)-15 induced both proliferation of human peripheral blood mononuclear cells (PBMCs) and IFN-γ secretion, and was used to successfully treat murine lung metastases by intranasal administration, leading to increased numbers of NK and cytotoxic T cells and macrophages, with the latter showing a polarization towards the antitumoral M1 phenotype [171]. Another transgenic *N. caninum* strain expressed and secreted a single-chain variable fragment fused to an Fc domain (scFv-Fc) binding to human programmed cell death ligand 1 (PD-L1). The scFv-Fc bound to PD-L1 on mouse and human tumor cells, blocked the programmed cell death protein 1 (PD-1)/PD-L1 pathway and induced T cell activity, antibody-dependent cellular phagocytosis, and cellular cytotoxicity [172]. Thus, *N. caninum* behaves as a live biotherapeutic capable of modulating both tumor growth and host immunity.

These findings place *N. caninum* within a broader emerging field where parasites are actively explored as immunostimulatory tools and sources for the development of anti-neoplastic strategies [173,174]. In parallel, engineered bacteria and other living microorganisms are being developed as programmable cancer immunotherapies and live biotherapeutic products. They incorporate genetic circuits that control tumor colonization, payload delivery, and safety profiles [175,176,177]. In this conceptual framework, *N. caninum* can be viewed as a potential Trojan horse-like programmable immuno-oncology platform. The parasite chassis can enter and perturb tumors while carrying defined therapeutic cargos and attenuation modules. Such a view bridges classical neosporosis control with the wider field of engineered live biotherapeutics.

## 9. Conclusions and Perspectives

Integrated control strategies remain paramount in mitigating the impact of *N. caninum* across diverse ecosystems. As shown in Argentina [66,71], selective breeding programs and embryo transfer have resulted in reduced vertical transmission and seroprevalence in dairy herds, providing a promising avenue for limiting reproductive losses in livestock. However, these programs require sustained commitment and consistent monitoring to ensure long-term success. Control strategies must also address the role of definitive hosts in environmental transmission, as well as the interface between farm and wildlife animals. The integration of “One Health” principles, linking human, animal, and environmental health, is crucial for the sustained success of control programs, particularly in regions with high human–livestock interactions.

The development of novel vaccines to be applied in cattle as the economically most important target host is important. These include live-attenuated and subunit vaccines, vaccines based on secretory components or exosomes, carbohydrate-based vaccines, and DNA or—based on the proven success against viral infections—mRNA vaccines [7,178]. Relevant vaccine targets in apicomplexan parasites are now being discovered by employing high-throughput approaches based on genomics, transcriptomics, and proteomics, together with techniques to genetically manipulate *N. caninum*. This will further improve our understanding of the biology of *N. caninum* and its interaction with the host, and will lead to the identification of determinants of virulence and triggers of immunity, thus contributing to the identification of novel targets for intervention [179]. Bridging the gap between immunological variability and vaccine development is essential for advancing effective intervention. In any case, a successful vaccine must elicit not only B cell responses, but also T-helper cell and cytotoxic T cell responses [65].

The chronic bradyzoite stage has a key role in transmission of *N. caninum* and should be more thoroughly investigated. The persistence of asymptomatic carriers represents a major challenge for control, and chronically infected animals maintain parasite populations within herds without exhibiting clinical signs, complicating detection and management [72]. In addition, bradyzoites pose significant barriers to treatment and immunological intervention, being largely resistant to chemotherapeutic agents and immune responses. Investigations on transcription factors that govern stage conversion in *N. caninum* could enhance our understanding of its chronic persistence, metabolic adaptation, and asymptomatic nature, which still remain critical knowledge gaps.

Overall, to overcome persistent obstacles in neosporosis control, a multidisciplinary approach is essential. Integrating genomic, proteomic, and transcriptomic data will provide a comprehensive understanding of host−parasite interactions, while international repositories of well-characterized *N. caninum* strains will enhance research consistency. The combined efforts of molecular research, therapeutic innovation, and collaborative surveillance frameworks will be pivotal in bridging the gap between laboratory discoveries and practical solutions.

## Figures and Tables

**Table 1:** General overview of the traditional laboratory methods used for the diagnosis of *N. caninum* infection.

Methods	Typical Samples	Sensitivity/ Specificity	Advantages	Pitfalls	REF
Histopathology Lesions consistent with neosporosis	Formalin fixed fetal brain, heart, skeletal muscle, and placenta	Dependent on sampling, supportive in combination with other methods	Fast, widely available, and relatively low cost	Not specific; the parasite may be sparse and not detected.	[24,25]
Immunohistochemistry Parasite antigens in tissue sections	Formalin-fixed tissues	High specificity and relatively low sensitivity among different laboratories	Detection of parasite in lesions	Not very sensitive at low infection burden. Performance depends on the operators and antibodies used.	[26]
Conventional or nested PCR Parasite DNA	Fresh, frozen, formalin-fixed tissues/sections	Most sensitive method (95–100%)	Possible also with low infection load; applicable to targeted lesions.	False positives from contamination; false negatives from inhibitors/poor extraction; DNA ≠ viability; sampling bias	[27]
Real-time PCR (qPCR) Parasite DNA quantification	Fresh, frozen formalin-fixed tissues/sections	Dependent on the platform/target; high analytical sensitivity and specificity	Very low detection limits and quantifying parasite burden; no post-PCR handling	Requiring specialized equipment and depending on sample quality. Different methods, targets, and cutoffs complicate cross-study comparisons.	[28]
IFAT (indirect fluorescent antibody test) Host antibodies	Serum, fetal fluids	High specificity + sensitivity reported	Serological reference method; good performance when well standardized	Requiring fluorescence microscopy + trained reader; lower throughput than ELISA; cutoffs differ by species/lab; subjective read-out	[29]
ELISA (enzyme-linked immunesorbent assay) Indirect or competitive detection of antibodies	Serum, milk (depending on kit/validation)	Multiple kits based on native or recombinant antigens available; differing sensitivities + specificities	High throughput, automatable, objective readout; good for screening/epidemiology and herd-level work	Kit-to-kit variation impairs cross-study comparisons; it needs local validation and appropriate cutoffs; positive serology does not prove disease.	[30]
Western blot Antibody binding to specific protein bands	Serum	Often used as a confirmatory test for corroboration of positive serology	High specificity; the ability to clarify ambiguous serology	Labor-intensive and low throughput, not ideal for primary screening; standardization is required	[31]
Agglutination tests (NAT, LAT, and DAT *) Antibodies	Serum	In multi-assay comparison in cattle, NAT sensitivity is lower than that of other methods.	Simple formats (esp. LAT), rapid and suitable for field studies	Often lower sensitivity than ELISA/IFAT; reagent production/standardization challenges	[29]
Isolation + in vitro culture Viable isolates	Fresh tissue homogenates (brain, placenta), body fluids inoculated into cultured cells	Not commonly used for diagnosis; generally low sensitivity	Definitive evidence of viable parasite; useful for research/strain work	Slow, specialized, biosecurity, and contamination issues; poor routine practicality	[32]
Bioassay Viable isolates	Fresh tissue homogenates inoculated into immunodeficient laboratory animals	Not used routinely; not typically reported for diagnostics	Very sensitive in expert hands; demonstrating parasite viability and infectivity	Ethical issues, slow, costly; not part of routine diagnostic practice	[33]

* DAT—direct agglutination test; NAT—*Neospora* agglutination test; LAT—latex agglutination test.

**Table 2:** Selected studies on subunit and live vaccines for prevention of *N. caninum* infection in cattle models. One study was carried out in ewes [118]. Vaccines contained either recombinant antigens or killed tachyzoite extracts, emulsified in different adjuvants [118,119,120,121,122,123,124,125], or were composed of attenuated live vaccines [126,127,128,129,130,131,132].

Antigen/Vaccine	Animal Model	Setup	Main Results	REF
NcSRS2 as DNA vaccine VR-NcSRS2PI formulated with a GM-CSF + Flt3L adjuvant; RecNcSRS2 peptides coupled with palmitic acid (lipopeptides LP 20–21 and LP34–36) and delivered once with Freund’s complete and once with an incomplete adjuvant.	Friesian Holstein cattle	Vaccination 3 × i.d. (neck region) with VR-NcSRS2PI DNA plus GM-CSF + Flt3L at 4-week intervals; 28 d.p.v. further 2 × immunization with LP 20–21 and LP 34–36 i.m.; 2 weeks apart. Lymphocyte proliferation assays, IFN-γ Elispot assays, and antibody analysis. No challenge infection.	Vaccination with DNA vaccine alone: no T-lymphocyte activation or IFN-γ secretion. Activation upon booster inoculation with NcSRS2-lipopeptides. NcSRS2-lipopeptide-immunized cattle significantly increased specific T-cell proliferation, numbers of IFN-γ-secreting PBMC, and IgG1 and IgG2a antibody levels.	[119,120]
RecNcGRA7 (*E. coli*) expressed as GST fusion protein and formulated in mannotriose-coated liposomes (M3-NcGRA7)	Holstein calves	Vaccination 2 × s.c., with 28-day intervals. 27 d.p.v., animals challenged i.v. with 10^7^ Nc-1 tachyzoites. Euthanized at 85–87 d.p.i.	NcGRA7-specific antibody production and IFN-γ production in PBMC in vaccinated group, pre-challenge. At 3 dpi, the body temperature and concentration of serum IFN-γ were higher in controls than in the immunized cattle. Two out of four were CNS PCR-negative in M3-NcGRA7-vaccinated cattle; all control cattle were CNS PCR-positive.	[121]
RecNcSAG1, NcHSP20 and NcGRA7 (*E. coli*) formulated as a mixture with ISCOMs	Angus heifers	Vaccination s.c. 2× with a mixture of all three antigens and ISCOMs; 3 weeks apart. Oestrus synchronization and natural breeding during 7 days. Challenge i.v. with 4.7 × 10^7^ Nc-1 tachyzoites at day 70 of gestation. Slaughter on day 104 of gestation.	Specific antibodies against all three antigens were elicited by the challenge, and their levels increased post-challenge. No differences in IFN-γ production were observed among the experimental groups at any time point. Transplacental transmission was determined in all fetuses by PCR, immunoblot, and immunohistochemistry.	[122]
Soluble *N. caninum* tachyzoite lysate fraction formulated in a soy-based aqueous adjuvant (sNcAg/AVEC)	Aberdeen Angus heifers	Artificially inseminated animals vaccinated s.c. 2× on days 65 and 75 of gestation; first challenge i.v. with 10^8^ Nc-1 tachyzoites (Nc1 strain) at 13 d.p.v.; second challenge at 146 d.p.v.	In vaccinated animals, IgM serum titers were induced as early as 7 d.p.v, followed by a switch to high avidity IgG, while naïve cattle elicited lower IgG titers, with a delayed kinetics. High systemic IFN-γ levels were induced after infection which did not interfere with pregnancy. No local or systemic adverse effects were detected. No efficacy results were reported.	[123]
RecNcPro, chimeric recNcPro fused to T-cell epitopes VSV glycoprotein G (rNcPro/G) or rNcPro/G formulated with alum hydroxide or a nanoparticulated AVEC enriched with TLR2 and TLR9 agonists (AVEC*plus*).	Adult heifers	Vaccination 2 × s.c. in the neck region, 21 days apart, collection of blood and sera at different d.p.v., and no challenge infection.	RecNcPRO alone induced only an IgM response, while rNcPRO/G-vaccinated animals switched to IgG1 after the booster. The vaccine formulated with rNcPRO/G and AVEC*plus* exhibited increased systemic IFN-γ and induced long-term recall B-cell responses.	[124]
Truncated recNcMIC1, NcMIC3, NcSRS2 and NcGRA7 formulated with cationic liposomes(Lip) + CpG-ODN	Holstein steers	Vaccination 2 × s.c. in the sub-scapular region, 21 days apart. Challenge i.v. with 10^8^ Nc-1 tachyzoites at 35 d.p.v.	Recombinant antigens formulated in Lip + CpG-ODN were highly immunogenic in cattle, and the IgG, IgG1, and IgG2 responses after challenge were enhanced. No efficacy results were observed.	[125]
Combination of recNcCyP and NcPro formulated in ImmunoMax SR Adjuvant System	Sheep	Vaccination 2 × i.m. in the neck, 4 weeks apart. Mating at 14 d.p.v. and challenged i.v. on day 90 of gestation with 10^6^ Nc-Illinois tachyzoites; abortions on days 120–148 of gestation, lambing on day 157.	Significant protection in the vaccinated group with 9 out of 13 ewes (69.2%) giving birth to live offspring, all ewes in the non-vaccinated group aborted. High-titer antibody responses were detected, including in colostrum. Live-born lambs and respective placentas *Neospora* PCR-negative; samples from aborted sheep all PCR-positive.	[118]
Live Nc Nowra tachyzoites	Holstein Friesian heifers	Vaccination i.v. once with live Nc Nowra prior to insemination, or s.c. 2× in the neck region with Nc Nowra tachyzoite lysate in VSA-3 or QuilA, 4 weeks apart, with insemination at 35 d.p.v. Challenge on 70 days of gestation with 10^7^ Nc-Liv tachyzoites.	High antibody titers in all groups, but strong cellular and IFN-γ responses only in the live-vaccinated group. Five out of six and five out of five cows in VSA-3 or QuilA groups (lysates), respectively, aborted; no abortion in the live-Nc Nowra group. Protection associated with high IFN-γ response.	[126]
Live Nc Nowra tachyzoites or frozen/thawed Nc Nowra tachyzoites	Beef heifers	Vaccination s.c. or i.v. with 10^7^ live Nc Nowra tachyzoites, or s.c. with 10^7^ frozen/thawed Nc Nowra. Artificial insemination at 63–75 d.p.v. At 139 d.p.v., confirmed pregnant heifers were challenged i.v. with 1.2 × 10^6^ *N. caninum* Nc-S197 tachyzoites. Euthanasia of all non-aborted heifers at 334 and 336 d.p.v.	Live vaccination s.c. or i.v. reduced abortion and the parasite detection in calves by PCR and serology after challenge from 55.6 to 85.2%; cryopreserved Nc-Nowra conferred 25.9% protection.	[127]
Live Nc-Spain 1H tachyzoites	Holstein Friesian heifers	Vaccination s.c. 2× at 4-week intervals with 10^7^ tachyzoites of the Nc-Spain 1H isolate in PBS over the left prescapular lymph node. Artificial insemination at 3 d.p.v.; challenge i.v. with 10^7^ live Nc-1 tachyzoites at 70 days or 135 days of gestation; either collection of aborted fetuses or euthanasia of born calves within 1 day after birth.	Challenge in early gestation: 50% protection against fetal death. Challenge at mid-gestation: no protection compared to the control. Calves from the immunized group had lower pre-colostral antibody titres, and strong antibody and IFN-γ responses were observed in the immunized heifers.	[128]
Live Nc-6 Argentina tachyzoites	Angus heifers	Vaccination 1 × i.v. with 6.25 × 10^7^ Nc-6 tachyzoites, or 2 × s.c. with Nc-6 tachyzoite-native antigen extract + ISCOMs, 3 weeks apart. Mating at 28 d.p.v.; challenge infection i.v. with 4.7 × 10^7^ Nc-1 tachyzoites at 70 d of gestation; slaughter at 104 days of pregnancy.	Increased antibody titers in both vaccinated groups from 21 d.p.v.; similar IFN-γ levels at week 13; all fetuses viable at necroscopy. Vertical transmission occurred in 1 of the 4 live-vaccinated fetuses and 3 of the 4 fetuses in the antigen extract-vaccinated group; 4 out of 4 fetuses in the non-vaccinated group were PCR-positive.	[129]
Live NcIs491 tachyzoites	Field trial with naturally infected pregnant dairy cows.	Routine breeding in one herd by artificial insemination. Selection of 520 seropositive heifers (titers 1:400 or above); 146 cattle vaccinated s.c. with 10^8^ freshly prepared NcIs491 tachyzoites in the anterior neck on days 125–150 of pregnancy; Follow-up of the dams for up to three consecutive pregnancies to study the long-term vaccine effect.	Lower abortion rate in vaccinated (16%) compared to non-vaccinated cows (26%), with 39% vaccine efficacy. No difference in seropositive offspring numbers in the two groups was identified; thus, vertical transmission was not prevented.	[130]
Frozen/thawed NcIs491 tachyzoites	Field trial with naturally infected pregnant dairy cows.	Routine breeding in 4 different herds by artificial insemination. Selection of 285 cows (titers 1:800 or above), 114 vaccinated 1 × s.c. with 2 × 10^8^ frozen/thawed NcIs491 tachyzoites on days 120–140 of pregnancy. The outcome of three consecutive pregnancies after a single vaccination was recorded in order to evaluate the long-term effect of vaccination.	Lower abortion rates in vaccinated animals at 3 out of 4 farms. Vaccine efficacy ranging from −19.8% to 75% at different farms, with an overall efficacy of 28.4% in all four farms and an overall efficacy of 58.2% in the three farms with positive results.	[131]
Live Nc-Argentina LP1 tachyzoites	Angus heifers	Vaccination 1 × s.c. with 10^6^ Nc-Argentina LP1 at 6 months of age; mating at 30 months of age; challenge (or not) on day 210 of gestation; evaluation of congenital infection after calving.	All animals live-vaccinated at prepubertal age underwent recrudescence as shown by serology. Congenital infection confirmed in 3 of 4 animals in the challenged group and in 2 of 4 animals in the vaccinated only group, showing that the use of live tachyzoites in young animals as a strategy to induce protection is neither safe nor effective.	[132]

i.d.—intradermal; i.v.—intravenous; s.c.—subcutaneous; i.m.—intramuscular; d.p.i.—days post-infection; d.p.v.—days post-vaccination; PBMC—peripheral blood mononuclear cell; GM-CSF—granulocyte-macrophage colony-stimulating factor; Flt3L—fetal liver tyrosine kinase 3 ligand; ISCOMs—immunostimulating complexes; AVEC—soy-based aqueous adjuvant; CpG-ODN—cytidine monophosphate guanosine oligodeoxynucleotides; TLR—Toll-like receptor; GST—glutathione-S-transferase.

## Data Availability

No new data were created or analyzed in this study. Data sharing is not applicable in this article.
